# Fractional erbium-doped yttrium aluminum garnet laser-assisted drug delivery: impact of triamcinolone acetonide formulation on drug permeation

**DOI:** 10.1007/s13346-024-01771-y

**Published:** 2024-12-24

**Authors:** Premrutai Thitilertdecha, Teerapat Wannawittayapa, Panyapat Buranaporn, Cyryl Rae Benjamine Santiago Rejuso-Kalbit, Rosalyn Kupwiwat, Poonsin Poungpairoj, Varangkana Tantithavorn, Nattawat Onlamoon, Woraphong Manuskiatti

**Affiliations:** 1 Siriraj Research Group in Immunobiology and Therapeutic Sciences, Faculty of Medicine Siriraj Hospital, Mahidol University, Bangkok, Thailand; 2 Department of Dermatology, Faculty of Medicine Siriraj Hospital, Mahidol University, 2 Wanglang Road, Bangkoknoi, Bangkok, 10700 Thailand

**Keywords:** Fractional ablative laser, Fractional Er:YAG laser, Triamcinolone acetonide, Topical bioavailability, Dermal absorption

## Abstract

Ablative fractional laser-assisted drug delivery has gained attention as a promising method for enhancing dermal drug absorption and improving therapeutic outcomes in dermatological conditions, particularly for hypertrophic and keloid scars. However, despite the growing number of clinical trials and case reports supporting its efficacy, there remains a scarcity of robust evidence on the topical bioavailability and dermato-pharmacokinetics of drugs in human subjects. This study aimed to examine the enhancement of triamcinolone acetonide (TAC) bioavailability following treatment with a fractional Erbium-Doped Yttrium Aluminum Garnet (Er: YAG) laser. Stratum corneum (SC) uptake and transport of TAC from 0.1% TAC cream and 10 mg/mL TAC solution/suspension with and without the laser pre-treatment were determined through tape stripping method for SC collection. TAC therein was quantified by an ultra-performance liquid chromatography coupled with photodiode array (UPLC-PDA) detection. TAC from both formulations without laser assistance was percutaneously absorbed within 6 h and TAC was delivered out from the solution to the SC remarkably higher. When the skin was pre-treated with the laser, permeability of TAC from the solution was escalated by 5 folds. TAC distribution profiles in the SC also confirmed this increased drug uptake, mainly the outer skin layers. On the other hand, amounts of absorbed TAC and their distribution patterns from the cream remained unchanged and low. No adverse events and unbearable pain were observed throughout the experiments. The fractional Er: YAG laser enhanced the dermal absorption of TAC, but this effect was confined to the solution formulation, with no significant improvement seen in the cream. This finding highlights the critical role that drug formulation plays in laser-assisted drug delivery. Moreover, factors such as drug selection, laser type, and optimal laser settings may also impact the efficacy of this approach and require further exploration.

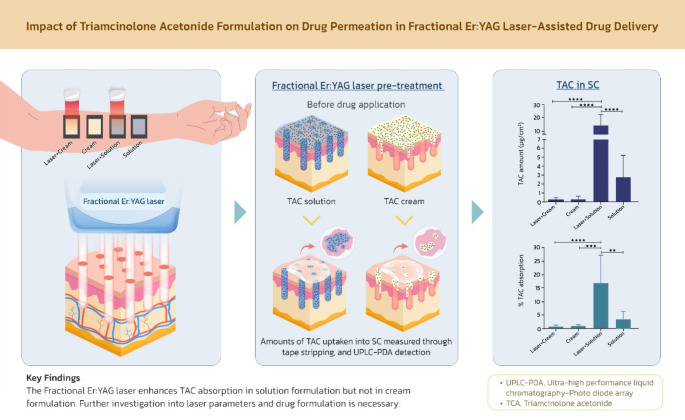

## Introduction

Laser therapy including pulsed dye laser (PDL), neodymium: yttrium-aluminumgarnet (Nd: YAG) laser, helium–neon (He-Ne) laser, as well as ablative and non-ablative fractional lasers, has emerged as a promising treatment for improving both the appearance and symptoms of keloid and hypertrophic scars (HTS) [1, 2]. Despite the advances in laser treatments, intralesional corticosteroid injections remain one of the most commonly used first-line therapies and adjunct treatments for keloids and HTS [3]. Corticosteroids function as potent glucocorticoids, suppressing the inflammatory response during the early stages of wound healing while enhancing collagen degradation by increasing collagenase activity and downregulating inhibitors such as α-1-antitrypsin and α-2-macroglobulin [4]. The current standard protocol involves administering a series of intralesional injections every 2–4 weeks until the scar is flattened, and its borders softened [5]. However, the drawbacks of this treatment modality include injection-related pain [6] and a higher risk of adverse effects, such as the development of telangiectasias, skin and subcutaneous fat atrophy, pigmentary changes (both hypo- and hyperpigmentation), skin necrosis, ulcerations, and even Cushing’s syndrome [7].

Recently, fractional laser-assisted drug delivery (FLADD) using ablative fractional lasers (AFXL) has emerged as a promising method for transcutaneous corticosteroid delivery, offering a reduced side-effect profile compared to conventional intralesional corticosteroid injections [8, 9]. This approach works by generating microscopic channels through the stratum corneum (SC), facilitating drug penetration while simultaneously creating microscopic injury zones that promote endogenous tissue remodeling [10]. Additionally, transcutaneous corticosteroid delivery has the advantage of minimizing systemic side effects while maintaining therapeutic concentrations of the drug within the target tissues.

Although multiple studies have demonstrated FLADD’s ability to enhance drug absorption and improve clinical outcomes [9, 11–16], concrete evidence regarding its efficacy in terms of drug bioavailability and dermato-pharmacokinetics in vivo in humans remains limited. To address these concerns, this study aimed to investigate the effectiveness of fractional Er: YAG laser in facilitating the transdermal delivery of triamcinolone acetonide (TAC) from two different formulations: a 0.1% TAC cream and a 10 mg/mL TAC solution/suspension. The study focused on the uptake and distribution profiles of the drug within the SC, while also assessing the safety of the treatment.

## Materials and methods

### Human subjects

Five healthy female volunteers aged between 23 and 37 years, with no history of skin disease, allergy to corticosteroids and hypertrophic scars/keloids as well as no visible skin abnormalities and no prior skin treatment in the preceding 4 weeks, were recruited to participation. Written informed consent was obtained from individuals prior to the study which was approved by the Siriraj Institution Review Board (SIRB) of the Faculty of Medicine Siriraj Hospital at Mahidol University, Thailand (Si 228/2023).

### Topical formulations

Two commercially available topical corticosteroid products including the 0.1% TAC oil-in-water cream (Aristocort^®^, Wyeth, Philadelphia, Pennsylvania, USA) and the 10 mg/mL TAC solution/suspension (Kanolone^®^, LBS Laboratory Ltd., Bangkok, Thailand) were selected based on their common used in real-world clinical practice for comparison between different dosage forms.

### Fractional Er: YAG laser treatment

A fractional Er: YAG laser (XS Dynamis, Fotona d.o.o., Ljubljana, Slovenia) was used to treat designated sites on the ventral forearms, operating at a pulse duration of 350 µs, an energy setting of 28 J/cm², and a density of 5%. To reduce pain and discomfort during the treatment, a commercially available air-cooling system (Cryo 6; Zimmer Aesthetics) was utilized. Based on schematic data provided by the manufacturer, the laser at these settings achieved an average vaporization depth of 87 μm and a coagulation depth of 10 μm. After laser treatment, a cream or solution was immediately applied to the treated areas and gently massaged into the skin for 2 to 3 min.

### Skin penetration study in human subjects

Dermal absorption of TAC in 2 different dosage forms was conducted over 2 days in which the first formulation (0.1% TAC cream) was applied to one arm on day 1 and the second formulation (10 mg/mL TAC solution) was applied to another arm on day 2. The ventral forearms were cleaned with an alcohol wipe and acclimatized for 30 min before treatment. Four skin sites including 1 control group (laser alone, site 1), 2 treated groups with laser assistance (laser with a cream or a solution, sites 2 and 3) and 1 treated group without laser assistance (a cream/solution alone, site 4) were delineated on each arm. Each treatment site was demarcated at 6 cm^2^ in area by a rectangular self-adhesive foam frame. One tape strip (Permacel J-LAR^®^) was discarded to remove SC disjunctum before treatment with drug application with and without laser assistance. A fingertip cut from a laboratory glove (nitrile and powder-free glove) was used to distribute the cream/solution over the demarcated area. Amounts of approximately 0.2 g of the cream or 50 µL of the solution were applied to sites 2 and 3 after laser treatment as well as site 4 without laser treatment. All sites were occluded and left for 6 h before drug removal and tape stripping. The actual weight of the formulation applied on each site was calculated based on the initial weight of the formulation and the different weight from the fingertip cut (i.e., before and after formulation distribution).

### Tape stripping and tape extraction

After the completion of 6-h application, excess cream/solution was gently wiped away and a new thin foam frame with 3 cm^2^ in area was placed over in the same position as the original frame. Only site 4 (a cream/solution alone) was measured for an initial value of transepidermal water loss (TEWL) using an AquaFlux^®^ evaporimeter (Biox System Ltd., London, UK). An adhesive tape strip (2.5 × 2.5 cm, Permacel J-LAR^®^, USA) was subsequently applied to the skin, pressed firmly down, quickly removed from the skin and measured again for the TEWL value. The procedure was repeated until TEWL values reached 4 times the initial value or when 30 strips had been taken. Data obtained from TEWL values were used to calculate SC thickness of individual subjects [17]. For other sites with laser treatment, the number was fixed at 15 strips [18] due to small bleeding caused by the laser treatment.

The mass of skin removed on each tape was determined through differences in weights before and after topical drug application using an ultramicrobalance (CUBIC ultramicrobalance model MSA2.7 S-000-DF, Sartorius, Göttingen, Germany). After that, the tapes were grouped for methanol extraction. The first and second tapes were individually analyzed and extracted in 1 mL methanol while the remaining tapes were combined into groups of 2–3 tapes and extracted in 1.5 mL methanol. The samples were left on the basic shaker overnight before the extracted solutions were withdrawn and filtered through a 0.45 μm nylon membrane for further TAC quantification. This in vivo methodology was previously proved to be reproducible and robust [19].

### Identification and quantification of TAC

Amounts of TAC were quantified using an ultra-performance liquid chromatography coupled with photodiode array (UPLC-PDA) method which was performed on a Waters Acquity™ UPLC system (Waters Corporation, Milford, MA, USA) and an Acquity UPLC™ BEH Shield RP18 column (1.7 μm, 100 mm × 2.1 mm i.d., Waters Corporation, Milford, MA, USA). The mobile phase was pumped at a flow rate of 0.45 mL/min and the column temperature was maintained at 50 °C throughout the analysis. The mobile phase was a combination of acetonitrile (A) and 0.05% aqueous trifluoroacetic acid (v/v, B) which was set as a gradient system of 30% A, 70% B for 3 min, 35% A, 65% B for 1 min and 30% A, 70% B for 1 min. TAC was detected at the wavelength of 242 nm from a Acquity UPLC^®^ PDA detector (Waters Corporation, Milford, MA, USA). All data acquired were processed by the Empower 2 software (Waters Corporation, Milford, MA, USA) and the complete validation of this analytical method was previously published [20].

### Statistical analysis

All statistical analyses were performed using GraphPad Prism^®^ version 7.02 (GraphPad Software, Inc., La Jolla, CA). Datasets were shown as mean or mean ± standard deviation (SD). A two-way ANOVA and Bonferroni post-tests were used to determine the statistical differences of the mean values among different groups. *P*-values ≤ 0.05 were considered as a statistical significance.

## Results

### Demographics of the subjects

A total of 5 healthy females aged between 23 and 37 years (an average of 28.7) were recruited in the study. All of them had similar demographic data as summarized in Table 1. All of them completed the 2-day experiments without drop-out.

**Table 1:** Demographic data of participants in this study (*n* = 5)

Characteristic	Value
Age (years), mean ± SD	28.7 ± 5.9
Gender, n (%) Female	5 (100%)
Fitzpatrick skin type, n (%) IV	5 (100%)
Skin diseases	None
Other underlying diseases	None
Current medication	None
Allergy	None

### The SC uptake of TAC

**Fig. 1:**
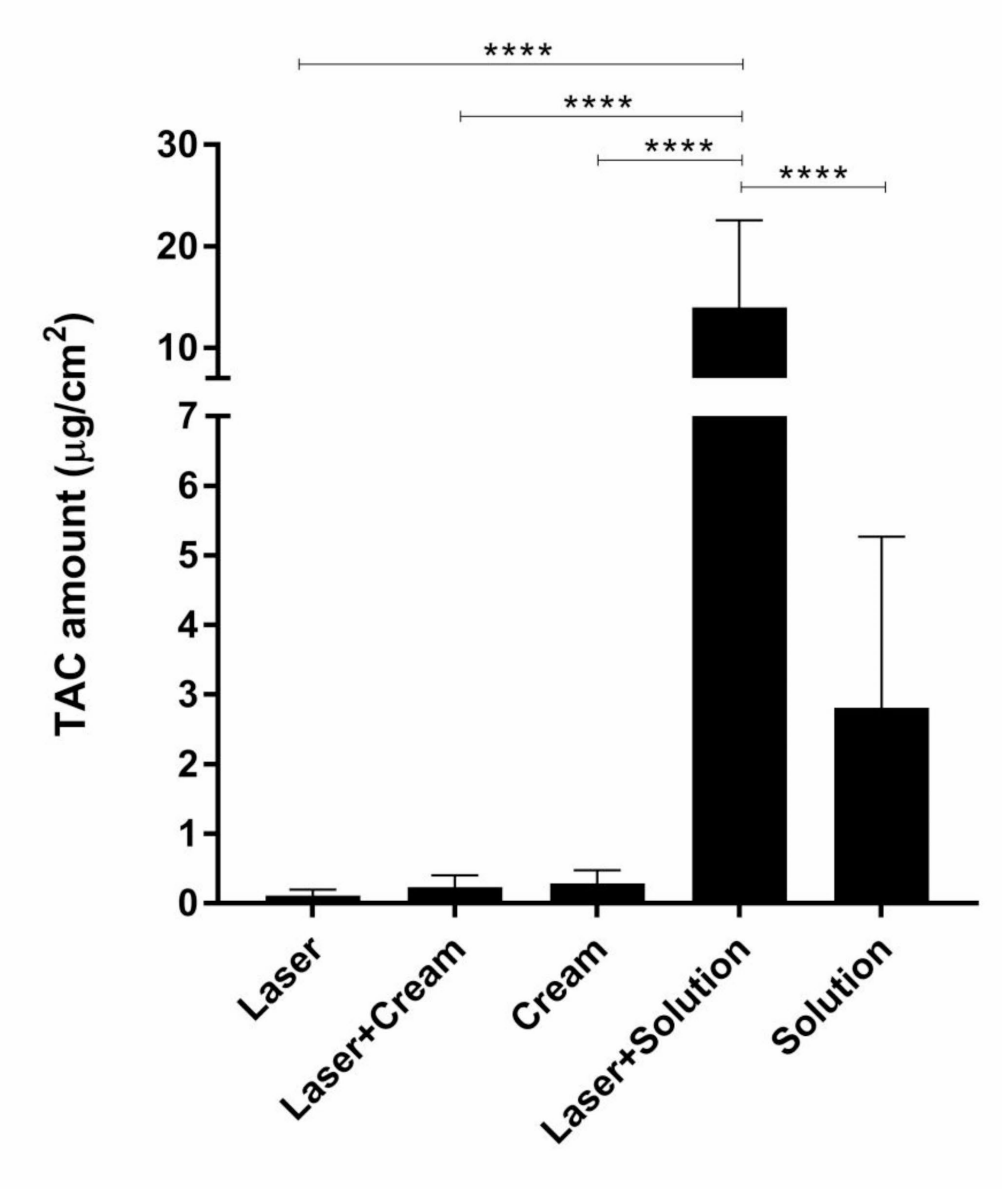
Total amounts of TAC in the SC after a 6-h application of a 0.1% TAC cream and a 10 mg/mL TAC solution with and without fractional Er: YAG laser treatment. All data are presented as mean ± SD (*n* = 10 for laser, laser + cream, and laser + solution groups; *n* = 5 for cream and solution groups)

Cumulative amounts of TAC penetrated into the SC of individual participants were determined at a 6-h post-application of a 0.1% TAC cream and a 10 mg/mL TAC solution with and without fractional Er: YAG laser treatment (Table 2; Fig. 1). The noise background for the negative control group, laser-treated site without TAC application was minimal to none. Even though the study’s analytical method was fully validated and shown to be reliable, this nevertheless happens frequently when utilizing UPLC-PDA detection [20].

Without the laser treatment, the penetration of the TAC solution was 9.7 times more than that of the TAC cream. On the other hand, with the laser-treated sites, the penetration of the TAC cream stayed nearly unchanged while that of the TAC solution significantly increased by 5 times (*p* < 0.0001). The two formulations differed much more as a result of this (60.9 folds, *p* < 0.0001).

**Table 2:** Amounts of TAC taken up into the SC after a 6-h application of a 0.1% TAC cream and a 10 mg/mL TAC solution with and without fractional Er: YAG laser treatment

Volunteer no.	TAC quantity (µg/cm^2^, average, *n* = 2)				
	Laser	Laser + Cream	Cream^a^	Laser + Solution	Solution^a^
1	0.15	0.06	0.32	26.95	7.20
2	0.00	0.22	0.06	9.33	1.82
3	0.02	0.51	0.56	15.97	1.87
4	0.03	0.15	0.21	11.14	1.64
5	0.20	0.23	0.30	6.61	1.50
*Average*	*0.08*	*0.23*	*0.29*	*14.00*	*2.81*
*SD*	*0.09*	*0.17*	*0.18*	*8.58*	*2.46*

^a^ Only 1 site was performed

### The influence of the donor concentrations on TAC absorption

**Fig. 2:**
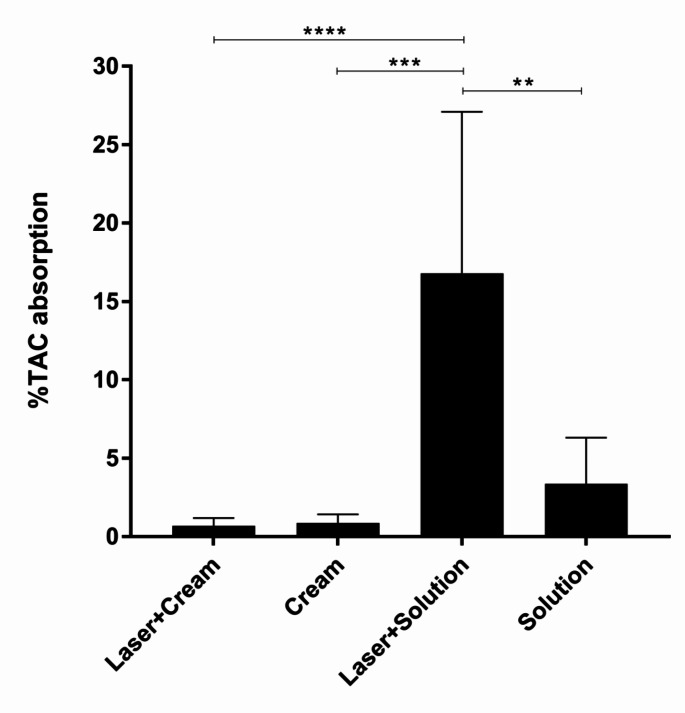
Percentages of TAC absorption in the SC after a 6-h application of a 0.1% TAC cream and a 10 mg/mL TAC solution with and without fractional Er: YAG laser treatment. All data are presented as mean ± SD (*n* = 10 for laser + cream and laser + solution groups; *n* = 5 for cream and solution groups)

The increased quantity of TAC solution penetrating the SC compared to the TAC cream can be attributed to the different loading amount of TAC at the beginning of the application. About 0.2 g of the TAC cream with a 0.1% concentration was applied, and 50 µL of the TAC solution with a 10 mg/mL concentration. Thus, there was a 2.5-fold difference between their initial TAC levels of 33.3 µg/cm2 and 83.3 µg/cm2, respectively. The volume and weight of the application are the standard amounts that were sufficient to cover the whole application area (6 cm2). Percentages of TAC permeated into the SC compared to their loading numbers were then calculated and shown in Table 3; Fig. 2. Even so, the absorption of TAC from the solution alone was compared to the cream (3.9 folds). The delivery of the laser-assisted TAC solution was noticeably high (16.8%) and significantly increased the permeability by 5 folds (*p* = 0.0022) and was still 24 times more than that of the delivery of the laser-assisted TAC cream (*p* < 0.0001).

**Table 3:** Percentages of TAC taken up into the SC after a 6-h application of a 0.1% TAC cream and a 10 mg/mL TAC solution with and without fractional Er: YAG laser treatment

Volunteer no.	% TAC absorption (average, *n* = 2)			
	Laser + Cream	Cream^a^	Laser + Solution	Solution^a^
1	0.17	0.96	32.33	8.64
2	0.67	0.17	11.19	2.18
3	1.53	1.68	19.16	2.24
4	0.45	0.64	13.38	1.97
5	0.67	0.90	7.93	1.80
*Average*	*0.70*	*0.87*	*16.80*	*3.37*
*SD*	*0.50*	*0.55*	*10.30*	*2.95*

^a^ Only 1 site was performed

### TAC distribution profiles in the SC layers

**Fig. 3:**
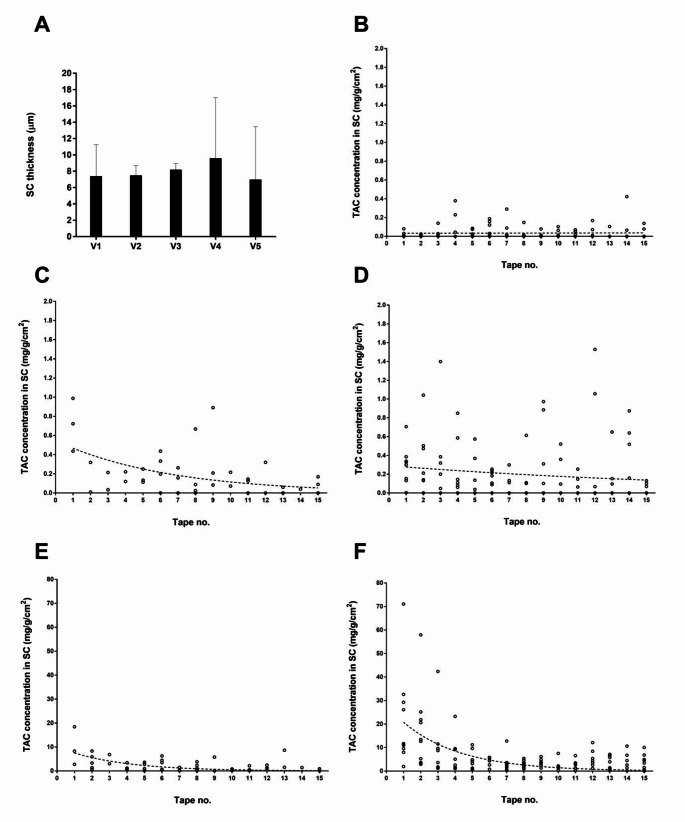
SC thickness and TAC concentration profiles in each SC layer subsequently removed by a tape strip after a 6-h application of a 0.1% TAC cream and a 10 mg/mL TAC solution with and without Fractional Er: YAG laser treatment from all volunteers (V1-V5). Total SC thickness of individual subjects (**A**) were expressed together with the TAC concentration profiles from sample groups of laser (**B**), cream (**C**), laser + cream (**D**), solution (**E**) and laser + solution (**F**). Values of SC thickness are presented as mean ± SD and datasets of TAC concentrations were individually presented (*n* = 10 for laser, laser + cream and laser + solution groups; *n* = 5 for cream and solution groups)

Total SC thickness of all participants was similar with an average of 7.9 ± 3.7 μm (Fig. 3A), reflecting the low variability in SC thickness between different individuals. Distribution profiles of TAC in the SC were usually expressed in the form of TAC concentration versus normalized SC depth; however, this study was not able to determine the SC depth due to the small bleeding caused by the laser treatment. The tape number was then considered as a representative instead and the penetration profiles of TAC as shown in Fig. 3C and E compared to the control samples (Fig. 3B, laser alone). The TAC cream that was not laser-assisted and those that was laser-assisted demonstrated rather consistent low concentrations across the SC (Fig. 3C and D, respectively). On the other hand, the TAC solution that was laser-assisted clearly improved particularly at the outermost SC layer in contrast to the TAC solution without laser assistance. (Fig. 3E). Consistent profiles were observed among 5 volunteers. Because of the sensitivity of the analytical method, as previously explained, the TAC content in the control sample (Fig. 3B) was the noise background at minimal to none.

### The safety of Fractional Er: YAG laser-assisted TAC delivery

Although multiple pinpoint bleeding spots appeared following the laser treatment and skin redness increased after tape stripping, the average pain scores recorded during the procedure ranged from 4.2 to 5.7 out of 10, indicating minimal to mild discomfort for participants. No subjects reported any serious adverse events or complications during or after the experiment. All participants achieved complete wound healing within one week, with no pigmentary changes, textural alterations, or scar formation.

## Discussions

The concept of fractional laser-assisted drug delivery (FLADD) has now been integrated into clinical practice for various applications, ranging from epidermal analgesia to treatment of nonmelanoma skin cancer, as it has been shown to enhance drug efficacy without increasing systemic side effects [8, 21]. Despite these promising results from clinical trials, the evaluation of this combined therapy in terms of topical drug bioavailability and dermato-pharmacokinetics remains limited, particularly in vivo. This study represents the first investigation in human subjects to demonstrate the effectiveness of the fractional Er: YAG laser in enhancing the delivery of the topical corticosteroid TAC, while also examining the impact of drug formulation on the amount of TAC absorbed into the SC and its distribution within the skin.

In this study, results show that fractional Er: YAG laser was able to increase TAC permeability from the solution by 5 folds, whereas no enhancement was observed when using the cream. This is possibly caused from differences in TAC partition from the formulation into the SC and TAC solubility in the skin which could be affected by co-solvents in the solution or surfactants in the cream [22]. Furthermore, the blood and fluids generated by the laser treatment may hinder the direction of TAC passive diffusion and alter the SC microenvironment, impacting its release rate and permeability. Another important factor is the initial TAC content in the applied formulations. The amount of TAC in the solution was 2.5 fold higher than that in the cream, so this could contribute to greater TAC uptake from the solution due to higher concentration gradient according to Fick’s law of diffusion.

In essence, our findings regarding the impact of topical formulations align with those of a prior clinical study that examined the use of fractional Er: YAG laser-assisted delivery of corticosteroids through an ointment containing 0.05% clobetasol propionate [23]. In that study, there was no significant difference in scar flattening between the area treated with clobetasol propionate and the area treated with petrolatum following laser treatment, even after two sessions and a 6-month follow-up. This implies that the steroid remained largely within the ointment base, failing to sufficiently penetrate the SC and reach the target treatment site. As a result, the improvement in scar appearance was likely attributed to the laser treatment itself rather than the corticosteroid.

Other important parameters contributing to the success of FLADD are the physicochemical properties of the drug, the topical application time frame after laser treatment and the duration of the application. TAC is a hydrophobic molecule with small size (molecular weight, MW = 434.5 Da) and intermediate octanol-water partition coefficient (log *P* = 2.5) which make it a good penetrant but poor in aqueous solubility (15–20 µg/mL) [24, 25], reflecting the TAC content in the formulation. The solution used in our study is at the concentration of 10 mg/mL which is assumingly at its saturation; therefore, the thermodynamic of TAC is at a maximum throughout the experiments. On the other hand, the solubility of TAC in the cream base is unidentified and whether it is at saturation is unknown. As microscopic channels created by AFXL gradually close over time, the appropriate time frame for topical drug application after fractional laser exposure needs to be established. Banzhaf et al. [26]. used a small hydrophilic compound to assess its enhanced penetration at several different time points up to 48 h after the laser treatment and demonstrated that the skin uptake was the maximum when topically applying the drug within the first 30 min after the laser exposure. Because there is no similar study available for hydrophobic compounds, in this study, topical application of TAC was administrated immediately after the laser treatment. Lastly, the application duration should be practical for both clinical and experimental settings while being long enough to allow for the detection and quantification of the penetrant. Pellanda et al. [27]. reported that TAC bioavailability could be quantified within 4 h post-application of 100 µg/cm² TAC solution in acetone. In the present study, TAC remained detectable even after a longer application time of 6 h, despite a lower drug loading content (33.3 and 83.3 µg/cm² in the cream and solution formulations, respectively). Additionally, the 6-hour application period was manageable for volunteers, allowing the experiment to be completed within a single day.

The use of FLADD to enhance topical drug delivery is not limited to TAC. Other topical corticosteroids, such as clobetasol propionate and betamethasone, as well as small hydrophilic and hydrophobic compounds, including 5-fluorouracil, hydroquinone, timolol, and 5-aminolevulinic acid, also show promise [11–15, 28]. In addition, other fractional ablative technologies, such as carbon dioxide (CO_2_) lasers, neodymium-doped yttrium aluminum garnet (Nd: YAG) lasers, and radiofrequency (RF) devices coupled with low-frequency acoustic pressure ultrasound (US), have been explored for their potential in enhancing drug delivery [12, 16, 26, 29]. However, no direct comparison has been made among these laser technologies to determine which is the most effective. It is also crucial to recognize that optimal laser settings greatly influence drug penetration. For instance, a previous in vitro study demonstrated that lower fluence settings of the Er: YAG laser were more effective for transdermal drug delivery [12]. Overall, laser-assisted drug delivery (LADD) shows significant promise, not only by enhancing drug absorption into the skin to reach target sites at therapeutic doses but also by improving clinical outcomes due to the laser’s inherent therapeutic effects.

## Conclusions

This study reinforces the efficacy and safety of fractional Er: YAG laser-assisted drug delivery for topical TAC, demonstrating increased uptake of TAC into the SC and enhanced drug distribution within the skin. The formulation and drug loading content play a critical role in determining permeability, with the solution/suspension proving superior to the cream in this combined therapy. It is likely that similar results would be observed with ointments and microparticles. Further research on the safety and efficacy of other corticosteroids and topical medications in combination with FLADD is warranted to explore new alternative treatment options for keloids and hypertrophic scars (HTS).

## Data Availability

All data generated or analyzed during this study are included in this published article.
